# A Randomized Controlled Trial of Concentrated ERP, Self-Help and Waiting List for Obsessive- Compulsive Disorder: The Bergen 4-Day Treatment

**DOI:** 10.3389/fpsyg.2019.02500

**Published:** 2019-11-15

**Authors:** Gunvor Launes, Kristen Hagen, Tor Sunde, Lars-Göran Öst, Ingrid Klovning, Inger-Lill Laukvik, Joseph A. Himle, Stian Solem, Sigurd W. Hystad, Bjarne Hansen, Gerd Kvale

**Affiliations:** ^1^Sørlandet Sykehus, Kristiansand, Norway; ^2^Department of Clinical Psychology, University of Bergen, Bergen, Norway; ^3^Department of Psychiatry, Molde Hospital, Molde, Norway; ^4^Bergen Center for Brain Plasticity, Haukeland University Hospital, Bergen, Norway; ^5^Department of Psychology, Stockholm University, Stockholm, Sweden; ^6^Department of Psychiatry, University of Michigan, Ann Arbor, MI, United States; ^7^Department of Psychology, Norwegian University of Science and Technology, Trondheim, Norway; ^8^Department of Psychosocial Science, University of Bergen, Bergen, Norway

**Keywords:** obsessive-compulsive disorder, OCD, ERP, RCT, B4DT, group therapy

## Abstract

**Background:**

The Bergen 4-day treatment (B4DT) is a concentrated exposure-based treatment for patients with Obsessive-Compulsive Disorder (OCD) delivered during four consecutive days. The B4DT has in a number of effectiveness studies demonstrated promising results as approximately 90% of patients gain reliable clinical change post-treatment and nearly 70% are recovered on a long-term basis.

**Methods:**

The current study is the first randomized controlled trial evaluating the effects of the B4DT. Forty-eight patients diagnosed with OCD were randomized to B4DT, self-help (SH), or waiting list (WL) with 16 patients in each condition. All participants randomized to the B4DT underwent the treatment without any attrition.

**Results:**

The B4DT yielded significantly better effects than control conditions on measures of OCD, depression, and generalized anxiety. The response rate (≥35% reduction of the individual patient’s pre-treatment Y-BOCS score) was 93.8% in B4DT, 12.5% in SH and 0% in WL, while remission rate (response criterion is fulfilled and the post-treatment Y-BOCS score is ≤12 points) was 62.5%, 6.3%, and 0%, respectively. Furthermore, patients who had received the B4DT, showed improved work- and social functioning. None of the patients treated with B4DT showed signs of deterioration. In comparison, one patient in the SH condition was in remission, and one showed significant clinical improvement, whereas the remaining showed no change.

**Conclusion:**

The results indicate that the B4DT is an effective treatment for patients suffering from OCD.

**Clinical Trial Registration:**

www.ClinicalTrials.gov, identifier NCT02886780.

## Introduction

Cognitive behavior therapy (CBT) including exposure and response prevention (ERP) is widely recognized as the treatment of choice for patients suffering from obsessive-compulsive disorder (OCD; Öst et al., 2015). A number of meta-analyses have shown that this treatment approach can be delivered successfully in a number of different formats including individual or group, in a concentrated form or weekly over several months (Abramowitz, 1998; Rosa-Alcázar et al., 2008; Öst et al., 2015).

However, patients with anxiety disorders rarely receive evidence based treatment (Shafran et al., 2009). Thus, self-help (SH) may be the only realistic treatment alternative for a substantial number of patients. In areas with low prevalence of competent therapists, the realistic alternative for many patients is the use of various SH manuals. Patients treated with SH can learn (e.g., from a workbook) about their disorder and how to apply treatment techniques to their own problems. SH can be delivered unassisted (pure SH) or guided (assisted by a clinician). Pearcy et al. (2016) published a meta-analysis of 18 studies (*N* = 1570) summarizing available RCT’s on SH for OCD, showing an average effect size of 0.51. Effect size for self-administered SH was smaller (*g* = 0.33) than for therapist-assisted SH (*g* = 0.91). Pearcy et al. (2016) concluded that there is a growing body of literature supporting the use of SH for OCD, but the authors also underlined the need for further studies in this area.

Even though group treatment approaches are effective, they tend to be associated with lower effect sizes as compared to individual treatments (Öst et al., 2015), which is in line with our clinic’s previous research, which indicates that more than 50% of the patients remained unchanged after a 12-session CBT group (Håland et al., 2010). Five to eleven years later, 62% of the patients were available for long-term follow-up interviews, and among these 40% were recovered and another 10% were improved, while 50% were unchanged (Sunde et al., 2017).

Recently, a highly concentrated CBT format including ERP, the Bergen 4-day treatment (B4DT), has shown promising results in a number of effectiveness studies (Havnen et al., 2014, 2017; Hansen et al., 2018, 2019). The B4DT can best be described as “individual treatment delivered in a group setting” because it is delivered to a group of 3–6 patients by the same number of therapists. The 1:1 ratio between patients and therapists ensures individually tailored exposure treatment, and the group setting provides the benefit of observing and working together with other patients.

More than 90% of the patients can expect relevant clinical change post-treatment, and nearly 70% are recovered on a long-term basis (Hansen et al., 2018, 2019). The approach is highly accepted by the patients, and there are essentially no dropouts. One of the strengths of the B4DT is that it was developed within an ordinary clinical setting, with low selection of patients, thus achieving high ecological relevance. Also, results of the B4DT has been replicated with new samples and new therapists (Havnen et al., 2017; Hansen et al., 2018), at new sites (Kvale et al., 2018; Launes et al., 2019) and in a different country (Davíðsdóttir et al., 2019). The B4DT has also been shown to significantly improve depressive symptoms as well as symptoms of generalized anxiety (Havnen et al., 2014; Hansen et al., 2018; Launes et al., 2019). However, the B4DT has not yet been evaluated in a randomized controlled trial (RCT).

The aim of the current study is to compare two exposure-based interventions for obsessive compulsive disorder, namely the B4DT and a SH ERP intervention based on Foa and Kozak’s (1997) approach in an RCT with waiting list (WL) as a control condition.

Based on the B4DT effectiveness studies, we expected the B4DT to be highly accepted by the patients. Also, we expected the B4DT to be superior to SH and to the WL condition on primary as well as secondary treatment outcome measures. Due to the inconclusive findings in the literature regarding self-administered SH, a clear hypothesis regarding the effect of this intervention seems premature. We also expected that the effects gained from the B4DT would be maintained at 6-month follow-up as seen in the previous effectiveness studies. Based on previous findings, we also expected significantly larger reduction in secondary outcome measures of depression, anxiety, work and social adjustment in the patients receiving B4DT, as compared to the other two conditions.

## Method

### Design and Randomization

The present trial is a randomized study where eligible OCD patients were randomized to one of three conditions, namely the B4DT, a 3-month SH intervention, or a 3-month WL. Based on ethical considerations, patients in the SH or in the WL condition were offered the B4DT after post-assessment if they still wanted treatment. Thus, there are no 6-month follow-up data on the patients in the SH- and WL condition.

Patient inclusion numbers were randomized (in blocks of six) using the online program Research Randomizer^1^ by an independent researcher at Karolinska Institutet, Stockholm, Sweden, to B4DT, SH or WL prior to study start. Directly after being included and receiving a study number, the patients were informed about their assignment. The enrollment was consecutive, so all patients referred to the clinic between August 2016 and September 2017, fulfilling the inclusion criteria and none of the exclusion criteria, were invited to participate in the study. If the patient agreed to participate, an informed consent was signed.

### Inclusion and Exclusion Criteria

Patients 18 years or older were eligible for inclusion in the study if they fulfilled the criteria of an OCD-diagnosis and had a Y-BOCS score of 16 or more; were fluent in Norwegian, and willing to sign the informed consent form. Exclusion criteria were: bipolar disorder, psychosis, ongoing substance abuse/dependence, hoarding behavior, intellectual disability based on previous medical history, eating disorder in need of medical attention, unwilling to refrain from anxiolytic drugs during the 4 days of treatment, ongoing suicidal ideation, unstable dose of antidepressant with recent dose-change within the last 4 weeks, and living more than 1.5 h drive by car/train form the treatment location. Also, patients who had undergone a previous full course of CBT treatment for OCD were excluded, due to an ongoing national trial targeting this group.

### Participants

The trial was conducted at an outpatient clinic in Kristiansand, part of the specialist health care in Southern Norway. Patients were referred from their general practitioner to a local outpatient clinic, and if their disorder was considered severe enough (Y-BOCS ≥ 16) to grant them treatment in the specialist health care, patients with OCD or suspected OCD, were referred to the specialized outpatient OCD-team at Solvang, Sørlandet Hospital. Eligible patients were informed orally and in writing about the study and the randomization procedure. The patients who declined participation were offered individual exposure-based CBT.

As part of standard care, potential participants were assessed using a standardized anamnestic interview which included the Mini-International Neuropsychiatric Interview (M.I.N.I.; Sheehan et al., 1998) and the Yale-Brown Obsessive Compulsive Scale (Y-BOCS; Goodman et al., 1989).

**FIGURE 1:**
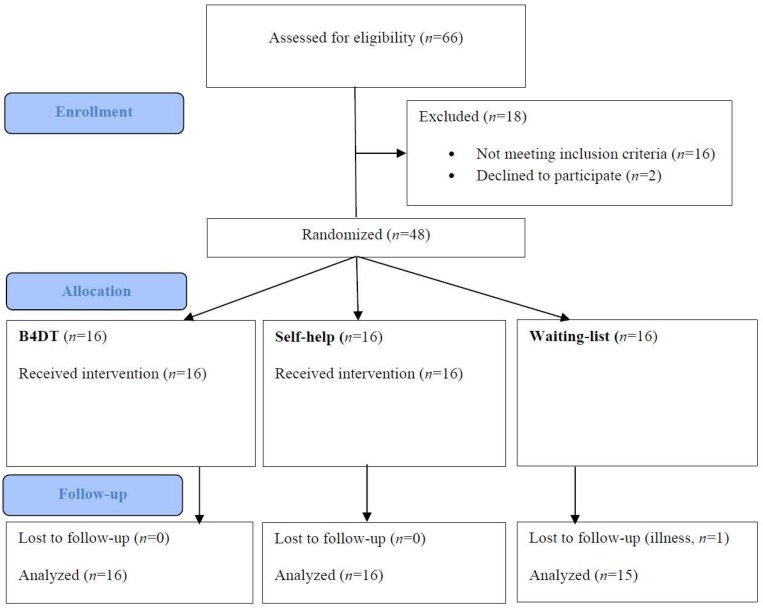
CONSORT flow chart. Reasons for exclusion were: other primary diagnosis (*n* = 3), sub-clinical OCD (*n* = 7), drug abuse (*n* = 2), inpatient (*n* = 1), and unstable medication (*n* = 3), declined to participate (*n* = 2).

During the inclusion period, 66 patients were considered for participation. 14 participants were excluded as they did not fulfill the inclusion criteria, or they fulfilled exclusion criteria for the study. One patient declined to participate in the study before signing the informed consent. One patient withdrew her consent within days after inclusion, as she was ambivalent about the concentrated group format. The other 50 proceeded to the diagnostic interview (see below). After this interview, one patient was excluded due to subclinical OCD and one because the SCID-interview revealed psychotic symptoms. See Figure 1 for flow chart describing patient flow throughout the study.

#### Background Data

Thirty-eight (79%) of the participants were female, and the mean age of the participants was 30 years, 31 (64%) were single. With respect to work status; 17 (35%) were working, 9 (19%) were students and 22 (46%) were on welfare benefits. Self-reported age of OCD onset was 15.52 (*SD* = 10.61) and the mean duration of OCD was 14.48 (*SD* = 10.15) years. There were no significant differences between the patients randomized to the three conditions regarding these variables. A summary of the participant’s background and diagnostic information is presented in Table 1. The sample had a wide range of OCD symptoms as indicated by scores on the DOCS-SF: 24 had obsessions related to contamination, 32 to being responsible for accidents, 19 to sexual, religious violence, or taboo related themes, 18 to symmetry/ordering, and 21 reported other contents of the obsessions. There were only 13 patients reporting just one type of OCD.

**TABLE 1:** Demographic and diagnostic characteristics.

	**Total**	**B4DT**	**SH**	**WL**	***χ^2^/F***	***p***
**Demographics**						
Age	30.35 (11.08)	33.35 (14.93)	27.75 (7.09)	30.06 (9.75)	0.99	0.38
Age at OCD onset	15.52 (10.61)	19.13 (14.25)	14.38 (8.72)	13.06 (7.20)	1.48	0.24
OCD duration	14.48 (10.15)	13.63 (8.62)	12.88 (10.15)	16.94 (11.64)	0.72	0.49
	***n* (%)**					
Female gender	38 (79)	14 (88)	13 (81)	11 (69)	1.77	0.41
Single	21 (44)	5 (38)	10 (63)	6 (31)	3.56	0.17
Work status					0.88	0.93
Student	9 (19)	4 (25)	3 (19)	2 (13)		
Employed	17 (35)	5 (31)	6 (38)	6 (38)		
Welfare benefits	22 (46)	7 (44)	7 (44)	8 (50)		
Psychotropics	21 (44)	5 (31)	8 (50)	8 (50)	1.52	0.47
**Comorbidity**						
# of disorders	2.15 (1.56)	1.50 (1.10)	2.56 (1.55)	2.38 (1.82)	2.23	0.12
No comorbidity	6	2	1	3		
GAD	15	5	5	5		
Major depression	17	1	9	7		
Dysthymia	9	0	6	3		
Panic/agoraphobia	15	2	7	6		
Social anxiety	9	3	4	2		
Specific phobia	5	4	1	0		
Insomnia	9	3	3	3		
PTSD	4	1	2	1		
ADHD	4	1	0	3		
Skin picking	4	0	2	2		
Other	9	3	2	4		

#### Pre-treatment OCD Severity

Forty-one (85%) patients had a severe OCD (Y-BOCS of 24–38), whereas seven (15%) had a moderate OCD (Y-BOCS of 18–23). Mean Y-BOCS scores pre-treatment were 27.17 (*SD* = 4.08). There were no significant differences in pre-treatment OCD-severity between the conditions (*p* = 0.702). Twenty-one patients (44%) were rated as having excellent insight, sixteen had good insight (33%), eight (17%) had fair insight, and three (6%) had poor insight, as measured by Y-BOCS item 11. There were no significant differences in insight between the conditions (*p* = 0.514).

#### Comorbidity

Forty-two (87%) of the patients had comorbid disorders (number of comorbid disorders ranged from one to six with a mean of 2.1, *SD* = 2.0). Fourteen patients (29%) had one comorbid disorder, 11 (23%) had two, six (12.5%) had three, seven (14.5%) had four, three (6%) had five, and one (2%) person had six comorbid disorders (for details see Table 1). There were no significant differences between the three conditions in total number of comorbid disorders, but there were significantly more patients with a comorbid depressive disorder in the SH- and waiting-list condition, compared to the B4DT-condition.

#### Pharmacological Treatment

Use of medication was registered at the initial interview. In the total sample, 12 (25%) used SRI/SSRI/SNRI, three (6%) used anti-psychotic medication, five (10%) had sleep medication prescribed for use when needed, and for three of the patients the sleep-medication was benzodiazepines; two used Ritalin (4%), and three (6%) used antiepileptic medication. There were no significant differences in the three conditions with regard to the use of psychotropic medication (see Table 1). Patients with and without psychotropic medication did not differ on Y-BOCS scores, *t*(46) = 0.035, *p* = 0.972, PHQ-9 scores, *t*(46) = 0.332 *p* = 0.332, or GAD-7 scores, *t*(45) = −0.239, *p* = 0.812, at pre-treatment. Patients on SSRI were encouraged to keep medication doses unchanged prior to and during the 4-day treatment period, and no changes in SSRI medication were reported at the post-treatment assessment. Patients were asked not to use any anxiolytics during the 4 days of treatment or to seek concurrent treatment for their psychological problems during the 4-day period. All patients reported that they adhered to this request.

### Assessment

The patients were asked to answer (online) a number of disorder-relevant questionnaires prior to randomization, and at post-treatment (except for WSAS which was administered only at pre-treatment and follow up for the B4DT group). For the patients who were randomized to the B4DT, these questionnaires were also administered at 3- and 6-month follow-up.

#### Diagnostic Interviews

After the patients were informed about the result of the randomization, they underwent a SCID-I interview (First et al., 2015) and a Y-BOCS interview conducted by an independent assessor by phone. Reliability and validity of conducting Y-BOCS interview by phone is supported by previous research (Baer et al., 1993). All participants were interviewed within a week prior to the intervention started. Post-treatment/waitlist assessment was done within 1 week after the condition ended. The OCD-module of the SCID and the Y-BOCS-interview were repeated at 3- and 6-month follow-up.

All Y-BOCS and SCID-I interviews for DSM-5 were conducted by specially trained and independent assessors. All the assessors were clinical psychologists, and had first received theoretical lessons in how to use the scales as well as practical instructions and video demonstrations which illustrated different scores on the items of the scales. To be qualified for the study, the assessors had received three videotaped full SCID-I interviews, and Y-BOCS interviews which they rated, and in order to proceed, the candidate had to demonstrate a minimum of 80% accuracy compared to an expert on 2/3 of the interviews. On Y-BOCS a maximum difference of ±2 points was employed. The assessor then had to perform three SCID-I interviews and Y-BOCS interviews with referred OCD-patients, which were videotaped and rated by a blinded expert (same requirements as above). All the phone interviews were taped. Adherence checks were performed on a random selection of 20% of the taped interviews. The checks were conducted monthly and feedback was given to the assessors. Inter-rater reliability both for OCD diagnosis before treatment (*κ* = 1.00) and Y-BOCS were excellent (intraclass correlation = 0.97).

### Therapists

In each treatment group, the patient to therapist ratio was 1:1. The groups were led by an expert on the 4-day format. All therapists had taken part in the national OCD-training program (Kvale and Hansen, 2014), or had documented equivalent training. Prior to participation, all therapists had participated in a minimum of two B4DT groups where they were independently rated as competent by two B4DT experts by using items of the OCD CORE competencies instrument (Steketee, 1993) relevant for the 4-day format. In order to become a leader of a B4DT group, it was required to have participated in a minimum of six B4DT groups and rated as competent by two independent assessors. Then these therapists functioned as leaders of a minimum of two B4DT group prior to the trial, and were rated as competent by two B4DT experts. Therapists included two psychiatrists, four psychologists and one psychiatric nurse, all with extensive experience as B4DT OCD-therapists.

#### Therapist Meetings Throughout the 4-Day Treatment

The patients were informed that the therapists worked as a team, and that the group leader would decide throughout the treatment which therapist would work with which patient during the next session. If considered feasible, each patient would work with more than one therapist in order to increase generalizability. Each day there were pre-scheduled brief therapist meetings throughout the day in order to ensure that the group leader and each therapist were informed about the progress and challenges for each patient. As a rule of thumb, the most experienced therapist worked with the most challenging patient, and also assisted and supervised in other exposure treatments when needed.

### Treatment

For a more comprehensive description of the treatment, see Launes et al. (2019).

#### The B4DT

##### Treatment preparation

The content and format of the B4DT was thoroughly described to eligible patients prior to inclusion. All were informed that the treatment needed to have their full attention, which meant that they could have no other appointments during the 4 days, and no appointments during the evenings of Day 2 and 3. If working, they were asked to take sick leave from work during the treatment. It was explained that it was essential to actively approach situations that triggered the OCD, and to learn new ways to deal with the anxiety and discomfort. It was underscored that the patients prior to the 4 days needed to prepare suitable exposure tasks, and were told that “the tasks that the OCD dislikes the most,” often were the ones that would create the largest change. Consequently, hierarchies where the exposure tasks are rank ordered based on the anxiety they are assumed to elicit, were not employed. After the patients were informed about the treatment, a modified version of the Borkovec and Nau (1972) “Treatment expectancy Scale” was applied. This contained four questions, namely (1) How logical does this treatment approach seem to you? (2) Would you recommend it to a friend? (3) What is the likelihood that you will fully dedicate the 4 days to the treatment and follow the treatment recommendations? (4) What do you think the likelihood is that you will benefit from the treatment? All questions were rated on a scale from 0 to 100. A rating below 70 was taken as an opportunity to explore and clarify misunderstandings. The week prior to the treatment, the leader called each patient in order to welcome them to the B4DT and to ensure that they were ready and had prepared relevant exposure tasks. The four questions addressing treatment expectancy were repeated. The call was scheduled to 15 min.

##### Day 1 psycho-education and exposure preparation (3–4 h)

The psychoeducation focused on the rationale behind ERP in the B4DT treatment, as well as important principles for change. Throughout the presentation, the OCD was externalized and described as a disorder that prevents them from living the life they want. It was underscored that the regulation of anxiety and discomfort through obvious or subtle avoidance and rituals maintain the symptoms, which means that in order to get rid of the OCD it is necessary to actively approach situations and stimuli that elicit OCD relevant anxiety and discomfort in order to learn to master it differently. In line with this anxiety was labeled “the raw material for change.” In addition, it was underscored that OCD typically demands a 100% certainty in situations when this is not an option, and that one goal was to learn to deal with a certain amount of uncertainty in the relevant situations. As a consequence, behavior experiments aimed at disconfirmation of catastrophic believes were not employed. Relevant exposure tasks were decided upon based on suggestions from the patients.

##### The LET intervention

One of the main features of the B4DT is to teach the patients to approach whatever elicits OCD-related anxiety or discomfort, and to help them systematically learn how to “**LE**an into **T**he anxiety” (LET-technique) instead of employing obvious or subtle avoidance. During the exposures, their task is to clearly demonstrate that they are doing something that is incompatible with practicing OCD-behaviors. It is underscored that if they are doing exposures and simultaneously try to neutralize their anxiety by following the demands of their OCD (keep their “OCD-project”), this will basically lead to discomfort without change. The patients are told that the OCD-project is often so integrated in their behaviors that they are typically not aware of the subtle avoidances and ritualizing, and that one of the main tasks for the therapist is to help the patient to be aware of these. If, for example, the task is to touch something that might trigger anxiety and discomfort due to potential risk of contamination, then hesitation, partially touching, and preventing spreading of the germs are seen as part of an OCD-project aimed at reducing anxiety and discomfort, which is to be replaced by an intention to “let go” of the control. Loop tapes can be used in these situations to maximize uncertainty, e.g., using repeated statements such as “I cannot be certain whether or not I got contaminated by touching all these objects.” Throughout the treatment it is systematically underscored that it is essential to first learn to do exposures with the correct technique (without subtle avoidance or rituals), and then to apply this approach to the tasks that “the OCD dislikes the most.” Whenever the patient starts any exposure exercise without clearly demonstrating that they are applying the LET-technique, the therapist will always suggest that the patient repeats an exposure that is done in accordance with the LET-technique. During the 2 days of exposure, therapists assisted each patient in practicing the LET-technique consistently whenever anxiety or discomfort is elicited, and the patient was encouraged to approach as many anxiety- or discomfort eliciting situations, contexts, and thoughts as possible.

##### Day 2 and 3

Day 2 started with summarizing the principles for the B4DT followed by an invitation to revise the individual exposure tasks in order to ensure that each patient had included all relevant tasks. The LET-intervention was then demonstrated. Throughout Day 2 and 3 (8 h each day), patients were engaged in individually tailored and therapist-assisted ERP in as many OCD-relevant settings as possible (including their home and sometimes their work) with continued self-administered practice in the evening, using the LET-intervention. The group met as a minimum in the morning, at lunch and in the afternoon where each patient shared their progress with the group and therapists. Specifically, they were asked to rate their own exposure performance on a scale from 1 to 6, where 6 indicated that they were “leaning fully into” the exposures. If they rated themselves less than 6, they were asked in which situations they were holding back, and what the “holding back” consisted of (e.g., hesitant exposures instead of starting right off), and they were encouraged to correct this during the next exposure. If considered useful, the patient reported progress to the therapist throughout the afternoon by brief text messages (e.g., “6” indicating that they were doing exposures in accordance with the LET-technique). In some cases, the therapist assisted the patient also in the evening in form of brief phone consultations. As a minimum, all patients reported back to the therapists once in the evening, typically at 9 pm with a text message stating their performance on the LET-intervention. If regarded as necessary, the therapist called the patients in order to troubleshoot exercises and to motivate patients to keep up with their LET exercises. Relatives and friends were invited to a psycho-educative meeting in the afternoon of day 3 (1.5 h).

##### Day 4: summarizing and relapse prevention

The last day was allocated to summarizing (“lessons learnt”) and planning self-exposures for the next 3 weeks. Focus was on teaching the patients how to be “their own therapists.” The patients were provided with written information covering procedures and times for upcoming assessments. At the end, the patients gave a structured feedback/evaluation of the treatment in the group setting.

##### Homework assignments during the first 3 weeks post-treatment

Participants logged their compliance (online) to the homework for 3 weeks after the 4-day group treatment. During this period, they did not have any contact with the clinic.

##### Follow-up visit at the clinic

Each patient had an individual follow-up meeting at the clinic about 12 weeks after the 4-day treatment was completed. The purpose of this appointment was to help the patient summarize their experiences following the B4DT, and if necessary, to refresh the principles of how to fight the OCD. No exposure therapy was done during this follow-up session.

#### The Self-Help Condition

The patients in the S-H condition received a SH book 1 week after the initial screening interview. The book is written by Foa and Kozak (1997), and covers both psychoeducation regarding OCD and “how-to-do” principles for ERP. Our recent meta-analysis (Öst et al., 2015) indicated that the range for SH interventions was 6–24 weeks, with a median of 12, and with 25% of the interventions lasting 9 weeks or less. The SH condition in the current study was 12 weeks from pre- to post-assessment, and there was no contact between patients and therapists during this period. Thus, this condition was a pure SH intervention (self-administered bibliotherapy).

### Adherence and Competency

Because the B4DT is delivered in a group setting with 3–6 therapists, it enables direct observation of therapists and group leader. There is also appointed a designated “second-in command,” who is responsible for ensuring that the protocol is followed, and that any deviances reported to the group leader are dealt with immediately. If the group leader did not comply with the protocol, the second in command was required to first notify the group leader and also inform the PI. No such reports were received.

The LET-intervention was demonstrated for the whole group of patients and therapists in the morning of Day 2, which enabled direct observation of adherence to the protocol. Deviances from the protocol were dealt with directly in the therapist meetings.

In addition to the psychoeducation which is presented Day 1; Day 2, 3 and also 4 start with a joint meeting for all. Also, everyone met for lunch and for a second meeting at approximately 3 pm Day 2 and 3. In the afternoon Day 3, there is a meeting for family and relatives. Deviations from the protocol are dealt with directly in the therapist meetings.

Directly after the treatment is completed, each therapist answers a brief questionnaire concerning whether the group was led in accordance with the protocol and with adequate competence, and whether each patient had been treated in accordance with the plan. Each question was rated “red” meaning not adequate/deviant; “yellow” meaning with some adjustments/deviations and “green” meaning adequate/in accordance with protocol. There were reported two “yellow” deviances. One addressing that a therapist had become ill the afternoon of Day 3 but the patient was adequately taken care of by another therapist. The other was addressing that the video camera did not work properly during all parts of the psychoeducation.

To enable external experts to assess if the protocol was followed, the psychoeducation, demonstration of the LET-intervention, all joint meetings between patients and therapists, all therapist meetings as well as the psychoeducation for the family/relatives were videotaped. Two independent assessors scored all parts of the three first groups and a 50% random selection of the rest. The assessment was done on a three-point scale: “red” = not in accordance with protocol, “yellow” = partly deviant and “green” = in accordance with protocol. All video recordings were scored “green” by both independent assessors.

### Measures

#### Yale-Brown Obsessive-Compulsive Scale (Y-BOCS; Goodman et al., 1989)

The Y-BOCS is regarded as the gold standard for assessing the severity of OCD symptoms. It consists of a symptom checklist covering obsessions and compulsions and a severity scale (Goodman et al., 1989). The severity scale comprises 10 items, rated on a 5-point Likert scale ranging from 0 (no symptoms) to 4 (severe symptoms). The total score ranges from 0 to 40. Y-BOCS has excellent inter-rater reliability and moderate to good internal consistency. Insight was measured by Y-BOCS item 11.

#### Structured Clinical Interview for DSM Disorders (SCID-I; First et al., 2015)

The SCID-I covers Axis I psychiatric disorders according to DSM-5.

#### The Patient Health Questionnaire 9-Item (PHQ-9; Kroenke et al., 2001)

The PHQ-9 is based on nine criteria for diagnosing depression in DSM-IV. Each item is reported on a four-point Likert scale (0, not at all; 3, almost every day), and the answers refer to the past 2 weeks. PHQ-9 has good psychometric properties and suggested cut-off scores for detecting major depressive disorder range between 8 and 11 (Titov et al., 2011; Manea et al., 2012).

#### The Generalized Anxiety Disorder 7-Item (GAD-7; Spitzer et al., 2006)

GAD-7 is based on the DSM-criteria for generalized anxiety disorder (American Psychiatric Association, 2013). Each item is reported on a four-point Likert scale (0, not at all; 3, almost every day), and the answers refer to the past 2 weeks. GAD-7 has good psychometric properties and suggested cut-off scores for identifying GAD range from 7 to 10 (Plummer et al., 2016; Rutter and Brown, 2017).

#### The Work and Social Adjustment Scale (WSAS; Mundt et al., 2002)

The WSAS is a short questionnaire measuring work and social adjustment. The scale consists of five items rated from 0 (not at all) to 8 (very severe), and higher score indicates higher impairment. The scale has good psychometric properties (Cella et al., 2011).

#### Obsessive-Compulsive Inventory – Revised (OCI-R; Foa et al., 2002)

The OCI-R is an 18-item self-rating scale, measuring six different symptom dimensions of OCD. The OCI-R total score has high test-retest reliability and high sensitivity to change (Foa et al., 2002; Solem et al., 2010).

#### Dimensional Obsessive Compulsive Scale Short-Form (DOCS-SF; Eilertsen et al., 2017)

The DOCS-SF is a self-report questionnaire adapted from the 28-item version of DOCS (Abramowitz et al., 2010). It consists of a symptom checklist covering obsessive and compulsive thoughts about “contamination,” “responsibility for harm, injury or bad luck,” “unacceptable obsessional thoughts,” “symmetry completeness and exactness.” For all dimensions, five items are rated (0–8 scale) on (a) time occupied by obsessions and compulsions, (b) avoidance behavior, (c) associated distress, (d) interference with daily functioning, and (e) difficulty disregarding obsessions and refraining from the compulsions. Items A and E are split into obsessions (0–4) and compulsions (0–4). The total score ranges from 0 to 40.

#### The Client Socio-Demographic and Service Receipt Inventory (CSSRI; Chisholm et al., 2000)

The CSSRI records socio-demographic data as well as previous treatment history.

#### Client Satisfaction Questionnaire 8 (CSQ-8; Larsen et al., 1979)

The CSQ-8 is an 8-item self-report scale on which patients report their level of satisfaction with the treatment they have undergone. The items are scored from 1 (very low satisfaction) to 4 (very high satisfaction), which give a total score from 8 to 32. The CSQ-8 is found to have good test-retest reliability and internal consistency (Nguyen et al., 1983).

### Treatment Completion, Response and Remission

Treatment completion was defined as attending all 4 days. Treatment response and remission were defined using a modification of the international consensus criteria (Mataix-Cols et al., 2016): *Response* is a ≥35% reduction of the individual patient’s pre-treatment Y-BOCS score. A patient is *remitted* if the response criterion is fulfilled and the post-treatment Y-BOCS score is ≤12 points. In addition, we will use the categories *deterioration* (≥35% increase of the individual patient’s pre-treatment Y-BOCS score) and *no change* (neither a deterioration nor a response).

### Statistical Analyses

We used hierarchical linear modeling (HLM) with random intercepts to compare the two exposure based interventions (B4DT and SH) with the WL control condition. These models estimate effects for change from pre-treatment to post-treatment. In addition, interactions between time (pre-treatment and post-treatment) and treatment condition can be used to identify differences between conditions. Statistically significant interactions were followed up by examination of simple effects, with Bonferroni corrections for multiple comparisons. This is recommended for the purpose of testing pairwise combinations of levels of the within-subjects factor (Maxwell and Delaney, 2014).

Similar HLM models were run for all primary and secondary outcome measures. As there were significant differences between conditions on PHQ-9, the pre-treatment score was included as a covariate in the HLMs. A HLM was also used to examine if the effect from the B4DT would be maintained at the 3-month and 6-month follow-ups. This model therefore involved the B4DT group only, and included four measurement points (pre-treatment, post-treatment, 3- and 6-month follow-up).

The total non-response to the Y-BOCS interviews and self-report measures used in this study was minimal. At pre-treatment, all participants provided complete responses to all measures. At post-treatment, 95.8% of the participants (*n* = 46) provided complete responses, and the amount missing data points totaled to 2.4%. Participants in the B4DT group also received Y-BOCS-interviews at 3-month and 6-month follow-up. At 3-month follow-up, 100% of the participants provided complete data, whereas 87.5% provided complete data at 6-month follow-up. Following the principle of intention to threat, all participants were included in the analyses, irrespective of missing data at any of the measurement points. Hierarchical linear models do not assume balanced data and are able to account for missing data on the response variable by using all available data on each participant, and under the *missing at random* (MAR) assumption, provide unbiased estimates (Schafer and Graham, 2002; Molenberghs et al., 2004). HLM with maximum likelihood estimation is often preferred over multiple imputation because it involves fewer decisions to be made by the researcher, is more efficient, and produces a deterministic result (Allison, 2012). Unlike multiple imputation, which involves introducing variability into the process, maximum likelihood is deterministic and will always produce the same results when applied to a given set of data.

Within group effect sizes were calculated using Cohen’s *d*, defined as (Mpre − Mpost)/SDpre. Controlled between group effect sizes were calculated post-treatment as: (Mean B4DT – Mean SH or WL)/pooled standard deviation. Cut-offs for interpreting effect sizes were: small (0.2), medium (0.5), and large (0.8). Fisher’s exact probability test (2-tailed) was used to compare differences between the treatment conditions on dichotomous variables.

### Ethical Approval

The study was reviewed and approved by the regional committee of ethics for human research (REK Vest-2016/794). The trial was pre-registered (ClinicalTrials.gov Identifier: NCT02886780) as a RCT comparing the Bergen 4-day concentrated exposure treatment (B4DT) to an exposure-based SH manual, and WL as the control condition.

## Results

### Primary Outcome Measure

Results for the primary and secondary outcome measures are presented in Table 2.

**TABLE 2:** Means (SDs) for Y-BOCS, GAD-7, PHQ-9, DOCS-SF, OCI-R, and WSAS.

**Measure**	**Treatment**	**Pre**	**Post**	**3-m FU**	**6-m FU**	**Effect size (*d*) within group**			**Effect size (*d*) between groups post-treatment**		
							
		***M* (SD)**	***M* (SD)**	***M* (SD)**	***M* (SD)**	**pre–post**	**pre-3-m FU**	**pre-6-m FU**	**B4DT vs. SH**	**B4DT vs. WL**	**SH vs. WL**
Y-BOCS	B4DT	26.75 (4.23)	10.90 (4.35)	8.56 (5.75)	9.17 (6.89)	3.75	4.30	4.16	2.57	3.86	0.51
	SH	27.88 (4.22)	24.63 (6.18)			0.77					
	WL	26.88 (3.93)	27.32 (4.14)			–0.11					
DOCS-SF	B4DT	26.56 (3.81)	13.44 (7.08)	10.69 (8.18)	12.00 (8.16)	3.44	4.17	3.82	1.65	1.87	0.10
	SH	28.69 (3.61)	24.63 (6.47)			0.57					
	WL	28.56 (6.29)	25.24 (5.43)			0.53					
OCI-R	B4DT	22.69 (12.42)	7.94 (5.94)	6.13 (5.48)	8.06 (8.16)	1.19	1.33	1.18	1.38	1.67	0.24
	SH	29.25 (15.29)	22.00 (13.14)			0.47					
	WL	27.69 (14.42)	25.19 (13.38)			0.17					
PHQ-9	B4DT	10.06 (4.60)	5.81 (4.37)	4.81 (3.97)	5.44 (3.77)	0.92	1.14	1.00	1.44	1.74	0.04
	SH	16.31 (5.69)	12.63 (5.06)			0.65					
	WL	12.50 (3.65)	12.48 (3.21)			0.01					
GAD-7	B4DT	10.69 (4.21)	6.81 (3.89)	5.00 (3.71)	6.68 (4.69)	0.92	1.35	0.95	1.24	1.24	0.04
	SH	13.81 (3.97)	12.44 (5.10)			0.35					
	WL	13.60 (4.85)	12.24 (4.81)			0.28					
WSAS	B4DT	15.13 (8.31)	−	6.19 (7.48)	6.38 (6.04)	−	1.08	1.05	–	–	–
	SH	19.38 (7.20)	18.19 (6.93)			0.17					
	WL	21.81 (7.16)	20.27 (6.51)			0.22					

*Y-BOCS, Yale-Brown Obsessive Compulsive Scale; GAD-7, generalized anxiety disorder-7; PHQ-9, patient health questionnaire-9; DOCS-SF, dimensional obsessive compulsive scale – short form; OCI-R, obsessive compulsive inventory – revised; WSAS, work and social adjustment scale.*

#### Change in OCD Symptoms

There was a statistically significant difference in the average change from pre-treatment to post-treatment in favor of the two intervention groups, χ^2^(2) = 138.17, *p* < 0.001. Compared to the WL group, both the B4DT group (−16.48, 95% CI [−19.79, −13.17], *p* < 0.001) and the SH group (−3.62, 95% CI [−6.89, −0.35], *p* = 0.026) had a greater decrease in their Y-BOCS scores from pre- to post-treatment. Simple effects at post-treatment showed that the mean Y-BOCS score was significantly lower for the B4DT group compared with the WL group (−16.24, 95% CI [−20.10, −12.37], *p* < 0.001) and the SH group (−13.05, 95% CI [−17.23, −8.87], *p* < 0.001). There was no statistically significant difference between the means of the SH and the WL groups at post-treatment (−3.19, 95% CI [−7.10, 0.72], *p* = 0.153). Effect sizes (ES) are presented in Table 2. The B4DT had large within-group as well as between-group ESs compared to both the SH- and the WL-condition. The SH-condition had a large within-group ES and a medium between-group ES compared to the WL-condition.

A separate HLM involving the B4DT group only was conducted to examine if the effects gained from the treatment would remain over the two follow-ups. Two planned comparisons (3-month follow-up vs. post-treatment; 6-month follow-up vs. post-treatment) showed that mean Y-BOCS scores did not significantly change from post-treatment to 3-month follow-up (−2.20, 95 CI [−5.31, 0.90], *p* = 0.22) or from post-treatment to 6-month follow-up (−1.39, 95 CI [−4.62, 1.85], *p* = 0.67).

The B4DT was highly accepted by the patients, as indicated by the proportion of patients that accepted to participate in the RCT (98%), and the fact that all patients completed the treatment.

#### Response and Remission

At post-treatment the response rate was 93.8% in B4DT compared to 12.5% in SH, and 0% in WL. The rates of remission were 62.5%, 6.3%, and 0%, respectively. A chi-square test yielded a significant χ^2^ (4) = 36.4, *p* < 0.0001, showing that the B4DT had significantly better clinical response than SH and WL, which did not differ from each other.

Table 3 displays the clinical improvement for the B4DT comparing post-treatment and 6-month follow-up. Nine of the 10 patients who were in remission post-treatment remained in that category at follow-up, whereas one had a small worsening to the response category. Even more interesting is that four of the five (80%) responders post-treatment had advanced to the remission category at follow-up, whereas one backed down to the no change category. These changes yielded a follow-up remission rate of 81.3% compared to 62.5% at post-treatment, a non-significant difference (McNemar’s test 2-tailed, *p* = 0.37).

**TABLE 3:** Clinical improvement for the B4DT post-treatment and at 6-month follow-up.

	**Status at 6-month follow-up**				
		
**Status at post-treatment**	**Remission**	**Response**	**No change**	**Deterioration**	****Total****
Remission	9	1	0	0	10 (62.5%)
Response	4	0	1	0	5 (31.3%)
No change	0	0	1	0	1 (6.3%)
Deterioration	0	0	0	0	0
Total	13 (81.3%)	1 (6.3%)	2 (12.5%)	0	16

*Treatment response was calculated based on the international consensus criteria (Mataix-Cols et al., 2016) which require a ≥35% reduction of the individual patient’s pre-treatment Y-BOCS score in order to be classified as a clinically relevant response. A patient is classified as remitted if, in addition to response, the post-treatment Y-BOCS score is ≤12 points.*

All patients in the WL and 14 of the patients (87.5%) in the SH condition still fulfilled criteria for an OCD-diagnosis according to the SCID-I interview at post-treatment assessment. In comparison, 13 (81.3%) of the patients in the B4DT condition no longer met criteria for an OCD-diagnosis. This was a significant difference between the B4DT and the WL condition (Fisher’s exact test, *p* < 0.001) and the SH condition (Fisher’s exact test, *p* < 0.001).

### Secondary Outcome Measures

#### Self-Reported OCD-Symptoms

HLMs were conducted to compare the treatment effect on self-reported OCD-symptoms, as measured by DOCS-SF and OCI-R. There was a statistically significant difference in the average change from pre-treatment to post-treatment in favor of the B4DT group for DOCS-SF, χ^2^(2) = 30.25, *p* < 0.001. The decrease for the B4DT group was significantly larger than for the WL group (−9.65, 95% CI [−14.10, −5.21], *p* < 0.001). The change from pre- to post-treatment for the SH group was not significantly different from the WL group (−0.59, 95% CI [−5.04, 3.85], *p* = 1.00). Simple effects at post-treatment showed that the mean DOCS-SF score was significantly lower for the B4DT group compared with the WL group (−11.30, 95% CI [−16.00, −6.60], *p* < 0.001) and the SH group (−10.27, 95% CI [−15.33, −5.22], *p* < 0.001). There was no statistically significant difference between the means of the SH and the WL groups at post-treatment (−1.02, 95% CI [−5.83, 3.78], *p* = 1.00).

Likewise, there was a statistically significant difference in the average change from pre-treatment to post-treatment in favor of the B4DT group for OCI-R, χ^2^(2) = 14.05, *p* < 0.001. The B4DT group reported a significantly larger decrease in mean OCI-R score from pre- to post-treatment than the WL group (−12.24, 95% CI [−19.64, −4.84], *p* < 0.001). The change from pre- to post-treatment for the SH group was not significant (−4.74, 95% CI [−12.14, 2.66], *p* = 0.30). Multiple between-group comparisons revealed that the mean for the B4DT group was significantly different from the WL group (−16.02, 95% CI [−26.65, −5.39], *p* < 0.01) but not from the mean of the SH group at post-treatment (−10.92, 95% CI [−22.56, 0.71], *p* = 0.07). The difference in means between the WL and SH groups at post-treatment was not statistically significant (−5.09, 95% CI [−16.01, 5.81], *p* = 0.79).

#### Self-Reported Depressive Symptoms and Generalized Anxiety

There was a statistically significant interaction between time and condition for PHQ-9, χ^2^(2) = 11.24, *p* < 0.001. Both the B4DT group (−4.22, 95% CI [−7.27, −1.17], *p* < 0.001) and the SH group (−3.66, 95% CI [−6.71, −0.61], *p* = 0.014) had greater decreases in PHQ-9 scores from pre- to post-treatment than the WL group. Simple effects at post-treatment showed that the mean PHQ-9 score was significantly lower for the B4DT group compared with both the WL group (−6.66, 95% CI [−10.40, −2.92], *p* < 0.001) and the SH group (−6.81, 95% CI [−10.52, −3.11], *p* < 0.001). There was no statistically significant difference between the means of the SH and the WL groups at post-treatment (0.15, 95% CI [−3.59, 3.90], *p* = 1.00). There was no statistically significant interaction between time and condition for GAD-7, χ^2^(2) = 3.47, *p* = 0.18. There was, however, a main effect of time, indicating a general decrease in GAD-7 scores for all groups (−2.38, 95% CI [−3.52, −1.24], χ^2^(1) = 16.74, *p* < 0.001).

#### Self-Reported Work and Social Adjustment

There was a statistically significant difference in favor of the B4DT group for WSAS (B4DT follow-up vs. SH and WL post-treatment), χ^2^(2) = 13.22, *p* < 0.001. The change for the B4DT group was significantly larger than the change for the WL group (−7.52, 95% CI [−13.00, −2.03], *p* < 0.001). There was no statistically significant difference between the SH and WL groups (0.23, 95% CI [−5.25, 5.71], *p* = 1.00). Multiple between-group comparisons showed that the mean WSAS score was significantly lower for the B4DT group compared with both the WL group (−13.42, 95% CI [−19.43, −7.41], *p* < 0.001) and the SH group (−9.98, 95% CI [−16.47, −3.50], *p* < 0.001). There was no statistically significant difference between the means of the SH and the WL groups at post-treatment (−3.43, 95% CI [−9.58, 2.71], *p* = 0.54).

#### Patient Satisfaction With Health Services

The patients in the B4DT-condition reported very high satisfaction, measured with CSQ-8 (possible range 8–32) post-treatment, *M* = 29.69, *SD* = 2.62, range 24–32). See Table 4 for further details.

**TABLE 4:** Post-treatment scores on Client Satisfaction Questionnaire 8 for patients treated with B4DT.

	**Satisfaction rating**			
	
**Item**	**1**	**2**	**3**	**4**
Quality of service	0	0	5	11
Kind of service	0	0	5	11
Met needs	0	0	7	9
Recommend to friend	0	0	2	14
Amount of help	0	0	5	11
Dealt with problems	0	0	5	11
Overall satisfaction	0	0	3	13
Come back	0	0	5	11

*N = 16. Items are scored on a Likert scale from 1 (low satisfaction) to 4 (high satisfaction).*

## Discussion

The aim of this study was to compare the clinical effects of the Bergen 4-day treatment for patients with OCD, to a SH intervention based on the Foa and Kozak (1997) treatment approach in an RCT with WL as the control condition. As expected, the clinical changes observed following the B4DT were superior to the SH-condition and the WL-control. More than 93% of the patients in the B4DT responded post treatment, and the remission rate was nearly 63%, which was increased to 81% at 6-month follow-up. Two of the patients who received SH, both with moderate OCD-symptoms initially, achieved remission after the intervention, whereas none of the patients in the WL-condition did so.

The B4DT was highly accepted by the patients, as measured by the proportion of patients that accepted to participate in the RCT (98%). Also, there was no attrition since all patients completed the treatment. The patients reported high satisfaction with the treatment as indicated by a mean score of 29.69 on a scale with a maximum of 32. It is also noteworthy that this scale specifically asks if this was the kind of treatment they wanted, and whether they considered the amount of treatment to be satisfactory. The B4DT also improved depressive symptoms, generalized anxiety symptoms, and work and social adjustment.

The results for the patients treated with the B4DT are basically the same as have been found in a number of uncontrolled effectiveness studies (Havnen et al., 2014, 2017; Hansen et al., 2018) conducted by the originators of the B4DT, and replicated at new sites (Kvale et al., 2018; Launes et al., 2019), as well as in a new country (Davíðsdóttir et al., 2019). The Norwegian uncontrolled studies yielded post-treatment Y-BOCS means between 9.0 and 10.9, and the present study had a mean of 10.9; the follow-up means ranged between 10.0 and 10.8, whereas the present study had 9.2. Regarding remission rate the uncontrolled studies varied between 72% and 77%, but the present study was somewhat lower at 63%. However, at follow-up the present RCT had a somewhat higher rate at 81%, compared to the uncontrolled studies’ range of 60–77%.

A comparison of the effects of B4DT with those in RCTs of standard (not concentrated) ERP in our meta-analysis (Öst et al., 2015) is of interest. In the meta-analysis, non-concentrated ERP yielded a remission rate of 48% both post-treatment and at follow-up 3–12 months later. Thus, the 63% post and 81% at follow-up (after 6 months) for B4DT in the present study are promising, but it would take an RCT directly comparing these formats to ascertain if the concentrated treatment is superior to treatment with weekly sessions.

There were no significant OCD-related pre-treatment differences between the patients randomized to the different conditions. However, depressive symptoms, including both major depression and dysthymia, were significantly higher in the SH- compared to the B4DT-condition, as indicated both by structured clinical interview and self-report.

The results indicated that the SH intervention was not superior to the wait-list condition, even though two patients remitted following SH. This finding is in line with previous findings by, e.g., Moritz et al. (2011) and Schneider et al. (2015) who also employed a SH intervention without any therapist assistance.

We believe that the patients in the current study are representative of the patients seeking treatment for OCD in specialist health care settings. The trial was conducted at an outpatient clinic were B4DT is given as standard treatment for OCD. Only two patients declined to participate in the study. Both patient characteristics and results are very similar to previous studies of B4DT conducted in the specialized health care setting (Havnen et al., 2014, 2017; Hansen et al., 2018, 2019). Nearly ninety percent were suffering from severe OCD and 87% of the patients had at least one co-morbid disorder.

A potential challenge and limitation of the present trial, is that we did not systematically record whether the patients actually read and used the SH manual. This is in line with previous trials (e.g., Vogel et al., 2014), and this decision was made in order to ensure that the patients randomized to SH did not have any therapist contact during the intervention. It should, however, be noted that two of the patients in the SH condition remitted, which is an indication that the SH book was adequate for these patients. The controlled effect size of SH vs. WL was *d* = 0.51. Unfortunately, there is no RCT of self-administered bibliotherapy of ERP in the literature, but two RCTs of predominantly SH with this content. (Tolin et al., 2007) found a *d* = 0.65 and Vogel et al. (2014) reported a *d* = 0.13. Thus, SH in the present study was at least as good as the mean ES in these two studies. As B4DT were found to be more effective than SH and waiting-list, further studies should compare the treatment to more effective treatments such as therapist assisted SH, face-to-face CBT, internet delivered CBT, or SSRI.

Another potential challenge is the fact that all patients ahead of randomization, were granted the B4DT post SH/WL if they experienced a need for it, which might have affected their motivation to actually use the SH program. This is a common ethical challenge in this kind of clinical studies, and in Norway, where all OCD-patients are offered evidence-based treatment; it would not have been ethically approved to not offer ERP to patients randomized to SH or WL.

Even though patients with a previous full course of ERP-treatment were not part of this study, the patients in the current trial did not differ in OCD severity; duration of the disorder; number of comorbid disorders, or amount of previous contact with the specialist health care as compared to the patients in the previous open trials. Although the study was sufficiently powered to detect significant differences between conditions, the relatively small sample size is a limitation when comparing results for the current study to related treatments.

With respect to dissemination and allegiance aspects of the treatment, issues such as an international manual, therapist training, and certification of teams need to be addressed in future studies. The current study did not include qualitative research on patient- and therapist perspectives on the treatments included. Future studies could also systematically investigate these experiences.

In conclusion, results add to the literature supporting the effectiveness of B4DT.

## Data Availability Statement

The datasets generated for this study are available on request to the corresponding author.

## Ethics Statement

The study was reviewed and approved by the regional committee of ethics for human research (REK Vest-2016/794). The patients/participants provided their written informed consent to participate in this study.

## Author Contributions

GK, L-GÖ, BH, KH, and GL contributed to the study design. GL, I-LL, IK, TS, and KH contributed to the data collection. All authors contributed to the statistical analysis, interpretation of the data, and drafting of the manuscript, and approved the final version.

## Conflict of Interest

The authors declare that the research was conducted in the absence of any commercial or financial relationships that could be construed as a potential conflict of interest.

## Footnotes

1 https://www.randomizer.org
